# The Medical Law—Its Enforcement

**Published:** 1881-02

**Authors:** 


### THE MEDICAL LAW. ITS ENFORCEMENT.

At the recent meeting of the Erie County Medical Society, held in the city, acting upon the suggestions contained in the admirable address of the retiring President, Dr. F. F. Hoyer, the Board of Censors were directed to take steps with a view of securing the enforcement of the medical law. There are many practitioners in this vicinity who have failed to register their names as the law requires. The Society, acting in its corporate capacity, proposes to make the law something more than a dead-letter upon the statute book, as many previous enactments have proven to be. We only counsel moderation in this matter, and expect good results to follow the Society's action.

